# Supplementary material for: Egg consumption and bone mass density among the elderly: A scoping review
Source: PLOS Glob Public Health. 2024 May 2;4(5):e0002519. doi: 10.1371/journal.pgph.0002519 (PMC11065246; doi:10.1371/journal.pgph.0002519)
Supplement: S1 Appendix — (DOCX) [file pgph.0002519.s002.docx]

**Appendix**

**Search Strategies**

Web of Science = 254

| **SN** | **Keywords** | **Search Strings** |
| --- | --- | --- |
| 1 | Egg Consumption OR Egg  intake | (TS=(Egg Consumption)) OR TS=(Egg intake) and Preprint  Citation Index (Exclude – Database) |
| 2 | BMD OR Osteoporosis OR OSteopeania | (((((((((((((((((((((((((((((((((((((((TS=(Bone mass density )) OR  TS=(Osteoporosis)) OR TS=(Osteopenia)) OR TS=(Bone Densities)) OR TS=(Density, Bone)) OR TS=(Bone Mineral Density)) OR TS=(Bone Mineral Densities)) OR TS=(Density, Bone Mineral)) OR TS=(Bone Mineral Content)) OR TS=(Bone Mineral Contents)) OR TS=(Osteoporoses)) OR TS=(Osteoporosis, Post-Traumatic)) OR TS=(Osteoporosis, Post Traumatic)) OR TS=(Post-Traumatic Osteoporoses)) OR TS=(Post-Traumatic Osteoporosis)) OR TS=(Osteoporosis, Senile)) OR TS=(Osteoporoses, Senile)) OR TS=(Senile Osteoporoses)) OR TS=(Osteoporosis, Involutional)) OR TS=(Senile Osteoporosis)) OR TS=(Osteoporosis, Age-Related)) OR TS=(Osteoporosis, Age Related)) OR TS=(Bone Loss, Age- Related)) OR TS=(Age-Related Bone Loss)) OR TS=(Age- Related Bone Losses)) OR TS=(Bone Loss, Age Related)) OR TS=(Bone Losses, Age-Related)) OR TS=(Age-Related Osteoporosis)) OR TS=(Age Related Osteoporosis)) OR TS=(Age-Related Osteoporoses)) OR TS=(Osteoporoses, Age- Related)) OR TS=(Metabolic Bone Diseases)) OR TS=(Bone Disease, Metabolic)) OR TS=(Metabolic Bone Disease)) OR TS=(Osteopenia)) OR TS=(Osteopenias)) OR TS=(Low Bone Density)) OR TS=(Bone Density, Low)) OR TS=(Low Bone  Densities)) OR TS=(Low Bone Mineral Density) and Preprint Citation Index (Exclude – Database) |
| 3 | Elderly or Aged | (TS=(Elderly)) OR TS=(Aged) |
|  |  |  |

**CINAHL Database**

**Search Outcome = 1**

| **SN** | **Keywords** | **Search Strings** |
| --- | --- | --- |
|  | **Egg Consumption OR Egg intake** | TI egg consumption OR TI egg intake |
|  | **BMD OR Osteoporosis OR Osteopeania** | TI bone mass density OR TI ( osteoporosis or bone density or bone loss ) OR TI ( osteopenia or low bone mass or low  bone density ) OR TI ( bone density or bone mineral density or bone strength ) |
|  | **Elderly or Aged** | TI elderly or aged or older or elder or geriatric or elderly  people or old people or old people or senior |
|  |  |  |

**PUBMED Database**

**Search Outcome = 6**

| SN | Keywords | Search Strings |
| --- | --- | --- |
| 1 | (Egg Consumption) OR (Egg intake) | (("ovum"[MeSH Terms] OR "ovum"[All Fields] OR "egg"[All Fields]) AND ("consumptions"[All Fields] OR "economics"[MeSH Terms] OR "economics"[All Fields] OR "consumption"[All Fields])) OR (("ovum"[MeSH Terms] OR "ovum"[All Fields] OR "egg"[All Fields])  AND ("intake"[All Fields] OR "intake s"[All Fields] OR "intakes"[All Fields])) |
| 2 | BMD OR  Osteoporosis OR Osteopeania | "bone mass density"[Title/Abstract] OR "Osteoporosis"[Title/Abstract] OR "Osteopenia"[Title/Abstract] OR "bone densities"[Title/Abstract] OR "density bone"[Title/Abstract] OR "bone mineral density"[Title/Abstract] OR "bone mineral densities"[Title/Abstract] OR "density bone mineral"[Title/Abstract] OR "bone mineral content"[Title/Abstract] OR "bone mineral contents"[Title/Abstract] OR "Osteoporoses"[Title/Abstract] OR "osteoporosis post traumatic"[Title/Abstract] OR "osteoporosis post traumatic"[Title/Abstract] OR ("Post-Traumatic"[All Fields] AND "Osteoporoses"[Title/Abstract]) OR "post traumatic osteoporosis"[Title/Abstract] OR "osteoporosis senile"[Title/Abstract] OR (("Osteoporosis"[MeSH Terms] OR "Osteoporosis"[All Fields] OR "Osteoporoses"[All Fields] OR "osteoporosis, postmenopausal"[MeSH Terms] OR ("Osteoporosis"[All Fields] AND "postmenopausal"[All Fields]) OR "postmenopausal osteoporosis"[All Fields]) AND "Senile"[Title/Abstract]) OR (("Senile"[All Fields] OR "seniles"[All Fields] OR "senility"[All Fields]) AND "Osteoporoses"[Title/Abstract]) OR (("Osteoporosis"[MeSH Terms] OR "Osteoporosis"[All Fields] OR "Osteoporoses"[All Fields] OR "osteoporosis, postmenopausal"[MeSH Terms] OR ("Osteoporosis"[All Fields] AND "postmenopausal"[All Fields]) OR "postmenopausal osteoporosis"[All Fields]) AND "Involutional"[Title/Abstract]) OR "senile osteoporosis"[Title/Abstract] OR "osteoporosis age related"[Title/Abstract] OR "osteoporosis age related"[Title/Abstract] OR "bone loss age related"[Title/Abstract] OR "age related bone loss"[Title/Abstract] OR "age related bone losses"[Title/Abstract] OR "bone loss age related"[Title/Abstract] OR ((("bone and bones"[MeSH Terms] OR ("Bone"[All Fields] AND "bones"[All Fields]) OR "bone and bones"[All Fields] OR "Bone"[All Fields]) AND "Losses"[All Fields]) AND "Age-Related"[Title/Abstract]) OR "age related osteoporosis"[Title/Abstract] OR "age related osteoporosis"[Title/Abstract] OR "age related osteoporoses"[Title/Abstract] OR (("Osteoporosis"[MeSH Terms] OR "Osteoporosis"[All Fields] OR "Osteoporoses"[All Fields] OR "osteoporosis, postmenopausal"[MeSH Terms] OR ("Osteoporosis"[All Fields] AND "postmenopausal"[All Fields]) OR "postmenopausal osteoporosis"[All Fields]) AND "Age-Related"[Title/Abstract]) OR "metabolic bone diseases"[Title/Abstract] OR "bone disease metabolic"[Title/Abstract] OR "metabolic bone disease"[Title/Abstract]  OR "Osteopenia"[Title/Abstract] OR "Osteopenias"[Title/Abstract] OR "low bone density"[Title/Abstract] OR "bone density low"[Title/Abstract] |
| 3 | Elderly Or Aged | "Elderly"[Title/Abstract] OR "Aged"[Title/Abstract] |

**SCOPUS Database**

**Search Outcome= 54**

| **SN** | **Keywords** | **Search Strings** |
| --- | --- | --- |
|  | **Egg Consumption OR Egg intake** | egg AND consumption OR egg AND intake |
|  | **Bone Mass Density** | bone AND mass AND density OR osteoporosis OR bone AND density OR bone AND loss OR bone AND mineral AND density OR bone AND strength |
|  | **Elderly or Aged** | elderly OR aged OR elder OR elderly AND people OR old AND people |
|  |  |  |
