# Supplementary material for: Egg consumption and bone mass density among the elderly: A scoping review
Source: PLOS Glob Public Health. 2024 May 2;4(5):e0002519. doi: 10.1371/journal.pgph.0002519 (PMC11065246; doi:10.1371/journal.pgph.0002519)
Supplement: Attachment — Submitted filename: PGPH-D-23-01926_reviewer ( New).docx [file pgph.0002519.s003.docx]

**PLOS Global Public Health**

# Egg Consumption and Bone Mass Density among the Elderly: A Scoping Review

--Manuscript Draft--

| **Manuscript Number:** | PGPH-D-23-01926 |
| --- | --- |
| **Article Type:** | Research Article |
| **Full Title:** | Egg Consumption and Bone Mass Density among the Elderly: A Scoping Review |
| **Short Title:** | Egg Intake and Elderly Bone Density: A Review |
| **Corresponding Author:** | Folahanmi T. Akinsolu Lead City University Ibadan, NIGERIA |
| **Order of Authors:** | Mobolaji T. Olagunju |
|  | Olunike R. Abodunrin |
|  | Ifeoluwa O. Omotoso |
|  | Ifeoluwa E. Adewole |
|  | Oluwabukola M. Ola |
|  | Chukwuemeka Abel |
|  | Folahanmi T. Akinsolu |
| **Keywords:** | Egg Consumption; Bone Mass Density; Elderly; Cholesterol; Aging and Mobility |
| **Abstract:** | Introduction  Eggs offer a range of essential nutrients that could potentially support skeletal health as individuals age. Maintaining bone density is crucial for reducing the risk of fractures and improving overall mobility and quality of life in later years. Understanding the potential benefits of habitual egg consumption on bone mass density among older people is essential, given that the natural decline in bone mass density occurs with age. This area of research has not garnered sufficient attention basically because of the mixed reactions and conflicting reports about the safety of egg consumption especially among the elderly hence the scoping review aims to systematically examine the existing literature to map the evidence regarding the association between habitual egg consumption and bone mass density in elderly individuals.  Methodology  The scoping review adhered to the Preferred Reporting Items for Systematic Reviews and Meta-Analyses Extension for Scoping Reviews (PRISMA-ScR) guidelines to ensure methodological rigor and transparency. Five electronic databases were searched for published pieces of literature. The review included letters, reviews, observational studies, and experimental studies, while the exclusion criteria were books, grey literature, and publications not in English.  Results  Out of an initial 315 studies found across five databases, 27 duplicates were removed. After screening, 283 studies were excluded as they did not meet the study eligibility criteria. Only two studies were finally included in the review, with two excluded due to focusing on eggshell powder's impact on elderly bone mass and one involving children.  Conclusion  Limited research on the link between egg consumption and bone mass density in the elderly highlights the need for further investigation. Concerns about cholesterol have overshadowed potential benefits. Given aging populations and bone health challenges, exploring eggs' role in preventing falls and fractures is essential for a proactive approach to elderly well-being. |

| **Additional Information:** |  |
| --- | --- |
| **Question** | **Response** |
| **Financial Disclosure**  Enter a financial disclosure statement that describes the sources of funding for the work included in this submission and the role the funder(s) played. This includes grants and any commercial funding of the work or authors.  This statement will be typeset if the manuscript is accepted for publication.  *Please review the* [*submission guidelines*](http://journals.plos.org/globalpublichealth/s/submission-guidelines#loc-financial-disclosure-statement) *and the instructions link below for detailed requirements and guidance.* | N/A |
| **Competing Interests**  On behalf of all authors, disclose any competing interests that could be perceived to bias this work.  This statement will be typeset if the manuscript is accepted for publication.  *Please review the instructions link below and PLOS Global Public Health’s* [*competing interests*](https://journals.plos.org/globalpublichealth/s/competing-interests) *policy to determine what information must be disclosed at submission.* | There is no conflict of interest. |
| **Data Availability**  Before publication, Authors are required to make fully available and without restriction all data underlying their findings. Please see our [PLOS Data](https://journals.plos.org/globalpublichealth/s/data-availability) [Policy](https://journals.plos.org/globalpublichealth/s/data-availability) page for detailed information on this policy.  A **Data Availability Statement**, detailing | Available in the manuscript |

| where the data can be accessed, is required at first submission. Insert your Data Availability Statement in the box below.  Please see the [data reporting](https://journals.plos.org/globalpublichealth/s/submission-guidelines#loc-data-reporting) section of our submission guidelines for instructions on what you need to include in your Data Availability Statement.  This statement will be typeset if the manuscript is accepted for publication.  *PLOS allows rare exemptions to address legal and ethical concerns. If you have legal or ethical restrictions, please use the box below to detail these in full sentences for the Journal team to consider.* |  |
| --- | --- |

Manuscript	[Click here to](https://www.editorialmanager.com/pgph/download.aspx?id=243142&guid=e8ae2adb-e71f-4921-8be4-4af43b8730f9&scheme=1)

[access/download;Manuscript;Manuscript_Akinsolu.docx](https://www.editorialmanager.com/pgph/download.aspx?id=243142&guid=e8ae2adb-e71f-4921-8be4-4af43b8730f9&scheme=1)

# 1 Egg Consumption and Bone Mass Density among the Elderly: A Scoping

2 **Review**

3

## 4 Mobolaji T. Olagunju1fj, Olunike R. Abodunrin1fj, Ifeoluwa O. Omotoso2fj, Ifeoluwa E.

5 **Adewole3&, Oluwabukola M. Ola3&, Chukwuemeka Abel3&, Folahanmi T. Akinsolu^3,4^*fj,**

6

## 7 ^1^Nanjing Medical University, China

8 **^2^Chongqing Medical University, China**

## 9 ^3^Lead City University, Ibadan

10 **^4^Nigerian Institute of Medical Research, Lagos**

11

12	*****[**Folahanmi.tomiwa@gmail.com**](mailto:Folahanmi.tomiwa@gmail.com)

13

## 14 ^¶^These authors contributed equally to this work.

15 **^&^These authors also contributed equally to this work.**

## 16 Abstract

17 **Introduction**: Eggs offer a range of essential nutrients that could potentially support skeletal
18 health as individuals age. Maintaining bone density is crucial for reducing the risk of fractures and
19 improving overall mobility and quality of life in later years. Understanding the potential benefits
20 of habitual egg consumption on bone mass density among older people is essential, given that the
21 natural decline in bone mass density occurs with age. This area of research has not garnered
22 sufficient attention basically because of the mixed reactions and conflicting reports about the safety
23 of egg consumption especially among the elderly hence the scoping review aims to systematically
24 examine the existing literature to map the evidence regarding the association between habitual egg
25 consumption and bone mass density in elderly individuals.
26 **Methodology**: The scoping review adhered to the Preferred Reporting Items for Systematic
27 Reviews and Meta-Analyses Extension for Scoping Reviews (PRISMA-ScR) guidelines to ensure
28 methodological rigor and transparency. Five electronic databases were searched for published
29 pieces of literature. The review included letters, reviews, observational studies, and experimental
30 studies, while the exclusion criteria were books, grey literature, and publications not in English.
31 **Results**: Out of an initial 315 studies found across five databases, 27 duplicates were removed.
32 After screening, 283 studies were excluded as they did not meet the study eligibility criteria. Only
33 two studies were finally included in the review, with two excluded due to focusing on eggshell
34 powder's impact on elderly bone mass and one involving children.
35 **Conclusion**: Limited research on the link between egg consumption and bone mass density in the
36 elderly highlights the	need	for further investigation. Concerns	about	cholesterol have
37 overshadowed potential benefits. Given aging populations and bone health challenges, exploring
38 eggs' role in preventing falls and fractures is essential for a proactive approach to elderly well-
39 being.

40

41 **Keywords**: Egg Consumption; Bone Mass Density; Elderly; Cholesterol; Aging and Mobility

## 42 Introduction

43 Eggs are often considered nutritional powerhouses, and their potential contribution to bone health
44 is increasingly recognized [1], attributed to the rich array of essential nutrients found in eggs that
45 play pivotal roles in improving bone strength and overall skeletal well-being[2].
46 Firstly, eggs contain Vitamin D, a vital nutrient known for its role in calcium absorption. Calcium,
47 in turn, is a cornerstone in bone mineralization and density[3]. Adequate Vitamin D intake ensures
48 that the body's calcium is effectively absorbed and utilized in bone-building, thus promoting bone
49 health[4]. Furthermore, eggs are a source of Zinc, a mineral crucial in supporting bone formation
50 and repair processes[5]. Zinc contributes to synthesizing collagen, a protein essential for
51 maintaining bone structure and integrity. Its involvement in bone health has been highlighted in
52 various studies[5].
53 Eggs also feature osteogenic bioactive components such as lutein and zeaxanthin. While these
54 compounds are better known for their role in eye health and reducing the risk of age-related
55 macular degeneration, their potential impact on bone health is emerging. By mitigating oxidative
56 stress and inflammation, lutein and zeaxanthin may indirectly support overall health, including
57 bone health[6,7].
58 It's worth noting that previous research primarily focused on individual nutrient components of
59 eggs, such as calcium [8], protein [9], and, vitamin D [10] about bone health. These nutrients are
60 critical for maintaining strong bones, and their presence in eggs underscores the potential benefits
61 of egg consumption [3,4,8–10]. Historically, whole eggs have faced scrutiny due to their high
62 cholesterol content, and concerns about their impact on cardiovascular health have been the subject
63 of research[11–14]. However, recent studies have challenged this notion, suggesting that moderate
64 egg consumption may not significantly increase the risk of heart disease [13,14]. Yet, the specific
65 associations between habitual egg consumption and bone health remain relatively uncharted in
66 nutrition research.
67 Peak bone mass, reached in the third or fourth decade of life[7,15], is a critical foundation for
68 skeletal health. However, as individuals age beyond this point, a consistent and natural decline in
69 bone mass density impacts both women and men[15,16]. This age-related reduction in bone mass
70 density plays a pivotal role in the increased susceptibility to vertebral fractures among the elderly
71 population (ref). Understanding the factors that influence bone health during aging is paramount,
72 and dietary habits are a contributing factor. Habitual egg consumption has emerged as a potential
73 dietary strategy to promote and maintain good bone health, particularly among older people [17].
74 The unique nutritional composition of eggs, including vital nutrients like Vitamin D, Zinc, and
75 protein, may play a significant role in supporting bone density and minimizing age-related bone

76	loss[18].

77 This scoping review aims to systematically examine the existing literature to map the evidence
78 regarding the association between habitual egg consumption and bone mass density in elderly
79 individuals. By synthesizing the available research, we can gain valuable insights into the potential
80 benefits of including eggs as a dietary component in promoting skeletal health during aging.

81

## 82 METHODOLOGY

83 The scoping review adhered to the Preferred Reporting Items for Systematic Reviews and Meta-
84 Analyses	Extension	for	Scoping	Reviews	(PRISMA-ScR)	guidelines [19]	to	ensure
85 methodological rigor and transparency.

86

## 87 Research Question

88 The following inquiry steered the review:
89 i. What evidence exists regarding the association between egg consumption and bone mass density
90 in older individuals?

91

## 92 Articles Identification

93 The initial search was conducted in June 2023 on five electronic databases: Scopus, CINAHL,
94 Web of Science, Medline, and PubMed. The search was performed using the following search
95 strategies enumerated in Appendix 1. No protocol was published for this review.

96

## 97 Eligibility Criteria and Article Selection

98 The literature obtained through database searches was imported into Rayyan's reference
99 management software. Duplicates were removed using the "duplicate items" function. Three
100 independent reviewers (OA, IO, and MT) conducted title and abstract screening, following the
101 eligibility criteria set for this review. A full-text review of the remaining publications was then
102 completed independently by five researchers (OA, IO, MT, BM, and FT). No attempts were made
103 to contact authors or institutions to find additional sources. Any published manuscript presenting
104 findings related to the association between egg consumption and bone mass density, English
105 publications, and full texts available for extracting all relevant information were considered for
106 study inclusion. The review included letters, reviews, observational studies, and experimental
107 studies, while the exclusion criteria were books and grey literature publications and publications
108 not in English.
109 109

## 110 Data Charting

111 Information on the paper identifiers (title, author, link), the country, year of publication, the study
112 aims, study design, study location, the quantity of egg consumption, frequency of egg consumption,
113 reported impact and effect size were extracted from the publications included in this review. The
114 extracted information from each publication was compiled and summarized in Table 1.
115 115

## 116 RESULTS

117 The initial search using the predefined search terms from five databases yielded 315 studies. From
118 the studies, 27 duplicates were removed, and the remaining 288 studies were screened. After
119 screening, 283 studies were excluded because they did not meet the eligibility criteria. Five articles
120 were sought for retrieval, of which three were excluded because two were studies on the impact of
121 eggshell powder on bone mass among older people, and the last was a study conducted among
122 children (Figure 1). Only two of the studies were included in the review. Table 1 shows the details
123 of the included articles addressing the association between habitual egg consumption and bone
124 mass density among older people.
125 125
126 126
127 127
128 128
129 129
130 130
131 131
132 132
133 Insert “Figure 1: Study Flow chart.”
134 134

## 135 Overview of Studies

136 The study conducted by Pujia et al. (2022) delves into the association between egg consumption
137 and bone health, particularly whole-body bone mineral density (BMD) and the T-score, a key
138 metric for diagnosing osteoporosis and assessing treatment effectiveness. In an aging population
139 of 176 individuals aged 65 years and older, the researchers aimed to unravel the potential impact
140 of egg consumption on bone density. Eggs were singled out due to their intriguing bioactive
141 compounds, which might have positive implications for BMD. The study unearthed a statistically
142 significant positive association between whole-body T-scores and egg consumption. Those who
143 consumed more eggs exhibited higher T-scores, suggesting better bone density. Gender and body
144 mass index (BMI) also influence bone health. Females had notably higher T-scores, and individuals
145 with higher BMI tended to have better bone density. Intriguingly, multiple fractures were
146 negatively associated with daily egg intake, implying that those who consumed more eggs were
147 less prone to experiencing multiple fractures. HDL-C levels were linked positively with multiple
148 fractures, indicating a potential role for cholesterol in bone health. This study provides novel
149 insights into the relationship between egg consumption and bone health in older people. It suggests
150 that whole eggs might positively impact bone density, potentially reducing the risk of osteoporosis
151 and fractures in older individuals. However, the study's cross-sectional nature limits its ability to
152 establish causation, and further research, ideally in the form of randomized controlled trials, is
153 needed to confirm these findings. If substantiated, this study could pave the way for whole eggs
154 as a viable dietary strategy for maintaining bone health in older people, addressing a crucial aspect
155 of overall well-being in aging populations.
156 The research conducted by Zeng et al. (2013) focused on exploring the potential link between the
157 consumption of red meat, poultry (with or without skin), and eggs and the subsequent risk of hip
158 fractures among elderly Chinese individuals. The study adopted a case–control design, with
159 meticulous matching resulting in 646 pairs of participants from Guangdong Province, a coastal
160 region in China. The study specifically targeted hip fractures, a significant concern among older
161 people due to their impact on mobility and overall well-being. By investigating the dietary habits
162 of these individuals, the researchers sought to identify any associations between food intake and
163 hip fracture risk. The study's results indicated that, on average, men consumed around 20.2 g/d of
164 the examined foods, while women's intake was slightly higher at 22.6 g/d. Upon analysis, the odds
165 ratio (OR) for the risk of hip fracture about red meat, poultry, and eggs consumption was calculated
166 at 0.99 (with a confidence interval of 0.63 to 1.56). This result suggests no conclusive evidence of
167 a substantial association between egg consumption and the subsequent risk of hip fractures among
168 the elderly participants. While the study's findings do not indicate an egg consumption and hip
169 fracture risk link, it is essential to consider the complexity of dietary patterns and potential
170 confounding factors that may influence such outcomes. Overall, the research contributes to the
171 ongoing discourse on nutrition and its impact on bone health, particularly in the context of the
172 elderly population.

Table 1: Characteristics of Included Studies

| **S/N** | **Title** | **Author (year of publication)** | **Study Aim** | **Publicatio n Type** | **Gender distribution** | **Study Participants** |
| --- | --- | --- | --- | --- | --- | --- |
| 1 | Relationship between osteoporosis, multiple fractures, and egg intake  in healthy elderly | Pujia et al. (2021) | To assess the association between eggs consumption and bone density in a population of the elderly. | Cross- sectional study | Female- 112  Male- 64 | Participants were recruited from February 2013 to August 2016, and this study was a secondary analysis  of baseline data obtained from a study named: “Effect of the Mediterranean Diet on cognitive function in the elderly.” |
| 2 | The association of red meat, poultry, and egg consumption with risk of  hip fractures in elderly Chinese: A case–control study | Zeng et al., (2013) | To examine whether the dietary intake of different types of red meat, poultry (with or without skin), and eggs are associated with a subsequent risk of hip fracture in a 1:1 matched case-control study with 646 pairs of elderly Chinese  from Guangdong Province. | Case- Control study | 646 case patients and 646 matched control subjects (484 female pairs and 162 male pairs) | the cases were hip fracture patients admitted consecutively to four hospitals: the First Afﬁliated Hospital of Sun Yat-sen University, Guangzhou Orthopedics Trauma Hospital, Guangdong General Hospital, and the Orthopedics Hospital of Baishi District in Jiangmen City, Guangdong province. Between  June 2009 and January 2013, 1281 patients aged 55 to 80 who had resided in Guangdong province for over 10 years were admitted with hip fractures diagnosed within 2 weeks of their potential enrollment into the study and conﬁrmed by X-ray image. |
|  |  |  |  |  |  | Controls were individually matched to the cases by sex and years. Controls were subject to the same inclusion and exclusion criteria as cases except for history of fracture. There were two sets of controls: 183 (28.3%) hospital controls were in-patients who were admitted to the above-mentioned hospitals and Zhongshan Ophthalmic Center within one week and could be matched to the cases; 463 (71.7%) community controls were apparently healthy community residents recruited  in the same cities |

Table 2: Study findings

| **S/N** | **Title** | **Author (Year of publication)** | **Reported Quantity of Egg Consumed** | **Frequen cy of Egg Consum**  **ption** | **Reported effect on BMD** | **Conclusion** |
| --- | --- | --- | --- | --- | --- | --- |
| 1 | Relationship | Pujia et al. | About 56% | Weekly | WB T-score correlated positively | This cross-sectional study provides |
|  | between | (2021) | of study |  | with daily egg consumption (*r* = 0.16; | evidence of a positive link between |
|  | osteoporosis, |  | participants |  | *P* = 0.027) | whole egg consumption and |
|  | multiple |  | consumed from 1 |  | Multiple fractures correlated | WB-BMD. If confirmed through |
|  | fractures, and |  | to 5 eggs/weeks, |  | negatively with egg intake (*r* = -0.39; | future randomized controlled trials, |
|  | egg intake |  | with an average |  | *P* = 0.014). | considering the high nutritional value |
|  | in healthy |  | weekly |  | WB T-score remained positively | of eggs, their economic accessibility |
|  | elderly |  | consumption of |  | associated with egg intake (B = 0.02; | on the marketplace, and recent dietary |
|  |  |  | one egg per |  | P = 0.02) at regression analysis | guidelines, the incorporation of whole |
|  |  |  | week. |  |  | eggs into the diet of the elderly could |
|  |  |  |  |  |  | have a significant public health |
|  |  |  |  |  |  | impact, including osteoporosis |
|  |  |  |  |  |  | prevention and fracture risk reduction. |
| 2 | The association | Zeng et al., | Mean intake for | Daily | No evidence of an association | our ﬁndings suggest that greater |
|  | of red meat, | (2013) | men = 20.2 g/d, |  | between egg intake and the risk of hip | consumption of fatty, but not lean, red |
|  | poultry, and egg |  | and 22.6 g/d for |  | fracture (OR = 0.99; 95% CI: 0.63, | meat and egg is associated with higher |
|  | consumption |  | women |  | 1.56) | risk of hip fractures. Further studies |
|  | with risk of |  |  |  |  | are needed to conﬁrm the beneﬁcial |
|  | hip fractures in |  |  |  |  | effect of lean poultry on the |
|  | elderly Chinese: |  |  |  |  | prevention of hip fractures. |
|  | A case–control |  |  |  |  |  |
|  | study |  |  |  |  |  |

## Discussion

The scoping review highlights that only two studies show the relationship between habitual egg consumption and elderlies' bone fracture risk. Both studies had contradictory findings, though the two studies used different study designs and had different study outcomes. This scoping review shows a noticeable gap in the existing literature concerning the potential relationship between habitual egg consumption and bone mass density in the elderly population. This scarcity of research underscores the need for further investigation to establish a clearer understanding of the potential causal relationship between these variables.

The few primary studies examining the association between egg consumption and bone health among the elderly draw attention to a gap that may have been created because of the concerns about the cholesterol content found in eggs and its link with the increased risk of cardiovascular diseases and in Italy, linked to a higher risk of overall mortality and mortality due to cancer[20], Spain[21], and China[22]. Yet the protein content of eggs, such as ovotransferrin, has bone- preserving proprieties through inhibition of the bone resorption process[23]. There is also suggestive evidence that carotenoids in egg yolk could prevent bone loss[24,25]. Egg consumption is also associated with dyslipidemia, a condition characterized by abnormal blood lipid levels. Eggs, a rich source of dietary cholesterol, have historically been of concern because of their impact on blood cholesterol levels. The relationship between egg consumption and dyslipidemia can vary among individuals and is influenced by genetics and overall diet[26,27].

With advancing age, the issue of bone health becomes increasingly critical due to the heightened susceptibility to bone frailty and the associated risk of falls and fractures, which can have severe consequences, especially among older people [17,28]. Recognizing this vulnerability, it becomes imperative to provide older people with comprehensive lifestyle and dietary recommendations that empower them to make informed choices to maintain their health and well-being[29]. Interestingly, emerging evidence suggests that eggs, often overlooked due to concerns about their cholesterol content, contain compounds that could contribute to bone development and enhance bone strength. Recognizing eggs' potential role in bone health signifies the importance of considering them as a dietary component for the aging population. While the potential cardiovascular risks of consuming cholesterol-rich foods like eggs have garnered significant attention, the flip side of the coin reveals a more nuanced story. Recent research has highlighted that some cholesterol in eggs may possess

protective qualities against dyslipidemia, a condition characterized by abnormal levels of lipids in the blood, intricately linked to cardiovascular health[30,31]. These contradictory findings emphasize the complexity of the relationship between egg consumption, cholesterol, and overall health, suggesting that a blanket assessment of eggs as detrimental may not be entirely accurate[32].

In the broader context of bone health, eggs emerge as a potential source of nutrients that could contribute to maintaining bone density and reducing the risk of fractures among older people [33]. As a rich source of protein, eggs provide essential amino acids that play a role in bone matrix formation and maintenance[2]. Additionally, eggs are a natural source of vitamin D, a vital nutrient that aids in calcium absorption and bone mineralization. Combining these nutrients makes eggs a unique dietary option that aligns with the nutritional requirements necessary to support bone health in aging individuals[34].

Addressing the current research gap concerning the relationship between egg consumption and bone mass density is essential for advancing our knowledge of bone health and advocating evidence-based dietary recommendations for older people. As the population ages, preserving mobility and reducing the risk of falls and fractures becomes increasingly paramount[29]. Therefore, adopting a comprehensive approach that includes dietary components like eggs, appropriate exercise, and other lifestyle adjustments could play a pivotal role in promoting healthier aging.

One of the strengths of this paper was assessing the effect of habitual bone consumption on bone mass density in a larger population than reported in a single study, which helps estimate effect size better and makes it easier to make recommendations to the broader population of older adults scientifically. One major limitation of the study is the dearth of studies on the subject of discourse, which would provide an excellent way to dispel myths and fads surrounding the habitual consumption of eggs with sufficient scientific evidence on the subject across a wider population. Also, the study is limited in the uniformity of study design across the studies included.

In conclusion, the limited research exploring the potential link between regular egg consumption and bone mass density, especially among older people, highlights the necessity for more extensive investigation. The reluctance to delve into this relationship may partly stem from concerns regarding cholesterol content, which has historically overshadowed the potential health benefits of eggs. Nonetheless, considering the elderly population's susceptibility to bone-related issues and

the urgency of taking proactive measures to prevent falls and fractures, it becomes imperative to examine the potential contributions of eggs to bone health. As research advances, a balanced assessment of how eggs impact bone and cardiovascular health should guide dietary recommendations. This approach acknowledges the complexity of the nutritional composition of eggs and their potential role in enhancing the well-being of the aging population.

## Acknowledgment

We extend our heartfelt gratitude to Prof. Morenike Ukpong for her invaluable contribution in reviewing the manuscript. Her keen insights and rigorous evaluation significantly enhanced the quality and rigor of this work. Her expertise and constructive feedback were indispensable in refining our manuscript, thereby advancing the discourse in this field. We also appreciate her time and dedication amidst her other professional commitments. Her willingness to engage and collaborate with us has enriched our research, and for that, we are sincerely thankful.

Conclusion:

Recommendation:

## References

[1] Wu J. Eggs as functional foods and nutraceuticals for human health. Royal Society of Chemistry; 2019.
[2] Rizzoli R, Biver E, Brennan-Speranza TC. Nutritional intake and bone health. The Lancet Diabetes & Endocrinology 2021;9:606–21.
[3] Turner AG, Anderson PH, Morris HA. Vitamin D and bone health. Scandinavian Journal of Clinical and Laboratory Investigation 2012;72:65–72.
[4] Ebeling PR. Vitamin D and bone health: epidemiologic studies. BoneKEy Reports 2014;3.
[5] Frassinetti S, Bronzetti GL, Caltavuturo L, Cini M, Della Croce C. The role of Zinc in life: a review. Journal of Environmental Pathology, Toxicology and Oncology 2006;25.
[6] Coheley LM, Kindler JM, Laing EM, Oshri A, Hill Gallant KM, Warden SJ, et al. Whole egg consumption and cortical bone in healthy children. Osteoporosis International 2018;29:1783– 91.
[7] Lu J, Shin Y, Yen M-S, Sun SS. Peak bone mass and patterns of change in total bone mineral density and bone mineral contents from childhood into young adulthood. Journal of Clinical Densitometry 2016;19:180–91.
[8] Cashman K. Calcium intake, calcium bioavailability and bone health. British Journal of Nutrition 2002;87:S169–77.
[9] Teucher B, Dainty JR, Spinks CA, Majsak-Newman G, Berry DJ, Hoogewerff JA, et al. Sodium and bone health: impact of moderately high and low salt intakes on calcium metabolism in postmenopausal women. Journal of Bone and Mineral Research 2008;23:1477–85.
[10] Winzenberg T, Jones G. Vitamin D and bone health in childhood and adolescence. Calcified Tissue International 2013;92:140–50.
[11] Qin C, Lv J, Guo Y, Bian Z, Si J, Yang L, et al. Associations of egg consumption with cardiovascular disease in a cohort study of 0.5 million Chinese adults. Heart 2018;104:1756– 63.
[12] Guo J, Hobbs DA, Cockcroft JR, Elwood PC, Pickering JE, Lovegrove JA, et al. Association between egg consumption and cardiovascular disease events, diabetes and all-cause mortality. European Journal of Nutrition 2018;57:2943–52.
[13] Krittanawong C, Narasimhan B, Wang Z, Virk HUH, Farrell AM, Zhang H, et al. Association between egg consumption and risk of cardiovascular outcomes: a systematic review and meta-analysis. The American Journal of Medicine 2021;134:76–83.
[14] Zhang F, Li J, Chang C, Gu L, Xiong W, Su Y, et al. The Association of Dietary Cholesterol from Egg Consumption on Cardiovascular Diseases Risk Varies from Person to Person. Journal of Agricultural and Food Chemistry 2022;70:14977–88.
[15] Bonjour J-P, Chevalley T, Ferrari S, Rizzoli R. The importance and relevance of peak bone mass in the prevalence of osteoporosis. Salud Publica de Mexico 2009;51:s5–17.
[16] Curtis E, Litwic A, Cooper C, Dennison E. Determinants of muscle and bone aging. Journal of Cellular Physiology 2015;230:2618–25.
[17] Martin FC. Frailty, sarcopenia, falls and fractures. Orthogeriatrics 2017:47–61.
[18] Martiniakova M, Babikova M, Mondockova V, Blahova J, Kovacova V, Omelka R. The role of macronutrients, micronutrients and flavonoid polyphenols in the prevention and treatment of osteoporosis. Nutrients 2022;14:523.
[19] Tricco AC, Lillie E, Zarin W, O’Brien KK, Colquhoun H, Levac D, et al. PRISMA extension for scoping reviews (PRISMA-ScR): checklist and explanation. Annals of Internal Medicine 2018;169:467–73.
[20] Ruggiero E, Di Castelnuovo A, Costanzo S, Persichillo M, De Curtis A, Cerletti C, et al. Egg consumption and risk of all-cause and cause-specific mortality in an Italian adult population. Eur J Nutr 2021;60:3691–702. https://doi.org/10.1007/s00394-021-02536-w.
[21] Zamora-Ros R, Cayssials V, Cleries R, Redondo ML, Sánchez M-J, Rodríguez-Barranco M, et al. Moderate egg consumption and all-cause and specific-cause mortality in the Spanish European Prospective into Cancer and Nutrition (EPIC-Spain) study. Eur J Nutr 2019;58:2003–10. https://doi.org/10.1007/s00394-018-1754-6.
[22] Xu L, Lam TH, Jiang CQ, Zhang WS, Zhu F, Jin YL, et al. Egg consumption and the risk of cardiovascular disease and all-cause mortality: Guangzhou Biobank Cohort Study and meta- analyses. Eur J Nutr 2019;58:785–96. https://doi.org/10.1007/s00394-018-1692-3.
[23] Shang N, Wu J. Egg white ovotransferrin attenuates RANKL-induced osteoclastogenesis and bone resorption. Nutrients 2019;11:2254.
[24] Miranda JM, Anton X, Redondo-Valbuena C, Roca-Saavedra P, Rodriguez JA, Lamas A, et al. Egg and egg-derived foods: effects on human health and use as functional foods. Nutrients 2015;7:706–29.
[25] USDA F. Unites States Department of Agriculture Foreign Agricultural Service, Global Agricultural Trade System (GATS), Forest products trade statistics. Access: [Http://Www](http://Www/) Fas Usda Gov/Gats/Default Aspx 2011.
[26] Jacobson TA, Maki KC, Orringer CE, Jones PH, Kris-Etherton P, Sikand G, et al. National Lipid Association recommendations for patient-centered management of dyslipidemia: part

2. Journal of Clinical Lipidology 2015;9:S1–122.

[27] Godos J, Micek A, Brzostek T, Toledo E, Iacoviello L, Astrup A, et al. Egg consumption and cardiovascular risk: a dose–response meta-analysis of prospective cohort studies. European Journal of Nutrition 2021;60:1833–62.
[28] Aspray TJ, Hill TR. Osteoporosis and the Ageing Skeleton. In: Harris JR, Korolchuk VI, editors. Biochemistry and Cell Biology of Ageing: Part II Clinical Science, Singapore: Springer; 2019, p. 453–76. https://doi.org/10.1007/978-981-13-3681-2_16.
[29] Izquierdo M, Merchant RA, Morley JE, Anker SD, Aprahamian I, Arai H, et al. International exercise recommendations in older adults (ICFSR): expert consensus guidelines. The Journal of Nutrition, Health & Aging 2021;25:824–53.
[30] Thomas MS, Calle M, Fernandez ML. Healthy plant-based diets improve dyslipidemias, insulin resistance, and inflammation in metabolic syndrome. A narrative review. Advances in Nutrition 2022.
[31] Pereira T. Dyslipidemia and cardiovascular risk: lipid ratios as risk factors for cardiovascular disease. Dyslipidemia-From Prevention to Treatment 2012;14:279–302.
[32] Charoenngam N, Shirvani A, Holick MF. Vitamin D for skeletal and non-skeletal health: What we should know. Journal of Clinical Orthopaedics and Trauma 2019;10:1082–93.
[33] Fratoni V, Brandi ML. B vitamins, homocysteine and bone health. Nutrients 2015;7:2176– 92.
[34] Puglisi MJ, Fernandez ML. The health benefits of egg protein. Nutrients 2022;14:2904.

**Appendix**

**Search Strategies Web of Science = 254**

| **SN** | **Keywords** | **Search Strings** |
| --- | --- | --- |
| 1 | Egg Consumption OR Egg  intake | (TS=(Egg Consumption)) OR TS=(Egg intake) and Preprint  Citation Index (Exclude – Database) |
| 2 | BMD OR Osteoporosis OR OSteopeania | (((((((((((((((((((((((((((((((((((((((TS=(Bone mass density )) OR  TS=(Osteoporosis)) OR TS=(Osteopenia)) OR TS=(Bone Densities)) OR TS=(Density, Bone)) OR TS=(Bone Mineral Density)) OR TS=(Bone Mineral Densities)) OR TS=(Density, Bone Mineral)) OR TS=(Bone Mineral Content)) OR TS=(Bone Mineral Contents)) OR TS=(Osteoporoses)) OR TS=(Osteoporosis, Post-Traumatic)) OR TS=(Osteoporosis, Post Traumatic)) OR TS=(Post-Traumatic Osteoporoses)) OR TS=(Post-Traumatic Osteoporosis)) OR TS=(Osteoporosis, Senile)) OR TS=(Osteoporoses, Senile)) OR TS=(Senile Osteoporoses)) OR TS=(Osteoporosis, Involutional)) OR TS=(Senile Osteoporosis)) OR TS=(Osteoporosis, Age-Related)) OR TS=(Osteoporosis, Age Related)) OR TS=(Bone Loss, Age- Related)) OR TS=(Age-Related Bone Loss)) OR TS=(Age- Related Bone Losses)) OR TS=(Bone Loss, Age Related)) OR TS=(Bone Losses, Age-Related)) OR TS=(Age-Related Osteoporosis)) OR TS=(Age Related Osteoporosis)) OR TS=(Age-Related Osteoporoses)) OR TS=(Osteoporoses, Age- Related)) OR TS=(Metabolic Bone Diseases)) OR TS=(Bone Disease, Metabolic)) OR TS=(Metabolic Bone Disease)) OR TS=(Osteopenia)) OR TS=(Osteopenias)) OR TS=(Low Bone Density)) OR TS=(Bone Density, Low)) OR TS=(Low Bone  Densities)) OR TS=(Low Bone Mineral Density) and Preprint Citation Index (Exclude – Database) |
| 3 | Elderly or Aged | (TS=(Elderly)) OR TS=(Aged) |
|  |  |  |

## Cinahl Database Search Outcome = 1

| **SN** | **Keywords** | **Search Strings** |
| --- | --- | --- |
|  | **Egg Consumption OR Egg intake** | TI egg consumption OR TI egg intake |
|  | **BMD OR Osteoporosis OR OSteopeania** | TI bone mass density OR TI ( osteoporosis or bone density or bone loss ) OR TI ( osteopenia or low bone mass or low  bone density ) OR TI ( bone density or bone mineral density or bone strength ) |
|  | **Elderly or Aged** | TI elderly or aged or older or elder or geriatric or elderly  people or old people or old people or senior |
|  |  |  |

**PUBMED Database Search Outcome = 6**

| **SN** | **Keywords** | **Search Strings** |
| --- | --- | --- |
| 1 | **(Egg Consumption) OR (Egg intake)** | (("ovum"[MeSH Terms] OR "ovum"[All Fields] OR "egg"[All Fields]) AND ("consumptions"[All Fields] OR "economics"[MeSH Terms] OR "economics"[All Fields] OR "consumption"[All Fields])) OR (("ovum"[MeSH Terms] OR "ovum"[All Fields] OR "egg"[All Fields])  AND ("intake"[All Fields] OR "intake s"[All Fields] OR "intakes"[All Fields])) |
| 2 | **BMD OR**  **Osteoporosis OR Osteopeania** | "bone mass density"[Title/Abstract] OR "Osteoporosis"[Title/Abstract] OR "Osteopenia"[Title/Abstract] OR "bone densities"[Title/Abstract] OR "density bone"[Title/Abstract] OR "bone mineral density"[Title/Abstract] OR "bone mineral densities"[Title/Abstract] OR "density bone mineral"[Title/Abstract] OR "bone mineral content"[Title/Abstract] OR "bone mineral contents"[Title/Abstract] OR "Osteoporoses"[Title/Abstract] OR "osteoporosis post traumatic"[Title/Abstract] OR "osteoporosis post traumatic"[Title/Abstract] OR ("Post-Traumatic"[All Fields] AND "Osteoporoses"[Title/Abstract]) OR "post traumatic osteoporosis"[Title/Abstract] OR "osteoporosis senile"[Title/Abstract] OR (("Osteoporosis"[MeSH Terms] OR "Osteoporosis"[All Fields] OR "Osteoporoses"[All Fields] OR "osteoporosis, postmenopausal"[MeSH Terms] OR ("Osteoporosis"[All Fields] AND "postmenopausal"[All Fields]) OR "postmenopausal osteoporosis"[All Fields]) AND "Senile"[Title/Abstract]) OR (("Senile"[All Fields] OR "seniles"[All Fields] OR "senility"[All Fields]) AND "Osteoporoses"[Title/Abstract]) OR (("Osteoporosis"[MeSH Terms] OR "Osteoporosis"[All Fields] OR "Osteoporoses"[All Fields] OR "osteoporosis, postmenopausal"[MeSH Terms] OR ("Osteoporosis"[All Fields] AND "postmenopausal"[All Fields]) OR "postmenopausal osteoporosis"[All Fields]) AND "Involutional"[Title/Abstract]) OR "senile osteoporosis"[Title/Abstract] OR "osteoporosis age related"[Title/Abstract] OR "osteoporosis age related"[Title/Abstract] OR "bone loss age related"[Title/Abstract] OR "age related bone loss"[Title/Abstract] OR "age related bone losses"[Title/Abstract] OR "bone loss age related"[Title/Abstract] OR ((("bone and bones"[MeSH Terms] OR ("Bone"[All Fields] AND "bones"[All Fields]) OR "bone and bones"[All Fields] OR "Bone"[All Fields]) AND "Losses"[All Fields]) AND "Age-Related"[Title/Abstract]) OR "age related osteoporosis"[Title/Abstract] OR "age related osteoporosis"[Title/Abstract] OR "age related osteoporoses"[Title/Abstract] OR (("Osteoporosis"[MeSH Terms] OR "Osteoporosis"[All Fields] OR "Osteoporoses"[All Fields] OR "osteoporosis, postmenopausal"[MeSH Terms] OR ("Osteoporosis"[All Fields] AND "postmenopausal"[All Fields]) OR "postmenopausal osteoporosis"[All Fields]) AND "Age-Related"[Title/Abstract]) OR "metabolic bone diseases"[Title/Abstract] OR "bone disease metabolic"[Title/Abstract] OR "metabolic bone disease"[Title/Abstract]  OR "Osteopenia"[Title/Abstract] OR "Osteopenias"[Title/Abstract] OR "low bone density"[Title/Abstract] OR "bone density low"[Title/Abstract] |

|  |  | OR "low bone densities"[Title/Abstract] OR "low bone mineral  density"[Title/Abstract] |
| --- | --- | --- |
| 3 | Elderly Or Aged | "Elderly"[Title/Abstract] OR "Aged"[Title/Abstract] |
|  |  |  |

## SCOPUS Database Search Outcome= 54

| **SN** | **Keywords** | **Search Strings** |
| --- | --- | --- |
|  | **Egg Consumption OR Egg intake** | egg AND consumption OR egg AND intake |
|  | **Bone Mass Density** | bone AND mass AND density OR osteoporosis OR bone AND density OR bo ne AND loss OR bone AND mineral AND density OR bone AND strength |
|  | **Elderly or Aged** | elderly OR aged OR elder OR elderly AND people OR old AND people |
|  |  |  |

Flow Chart	[Click here to access/download;Figure;Figure 1.jfif](https://www.editorialmanager.com/pgph/download.aspx?id=233328&guid=d3fcf8b4-9c92-4b97-a224-020ef270ee6d&scheme=1)

Supporting Information

[Click here to access/download](https://www.editorialmanager.com/pgph/download.aspx?id=243141&guid=4979e50c-1696-44e0-8b04-ba5aa0c70111&scheme=1)

[**Supporting Information**](https://www.editorialmanager.com/pgph/download.aspx?id=243141&guid=4979e50c-1696-44e0-8b04-ba5aa0c70111&scheme=1)

[PRISMA-ScR-Fillable-Checklist_Akinsolu.docx](https://www.editorialmanager.com/pgph/download.aspx?id=243141&guid=4979e50c-1696-44e0-8b04-ba5aa0c70111&scheme=1)
