# Supplementary material for: Egg consumption and bone mass density among the elderly: A scoping review
Source: PLOS Glob Public Health. 2024 May 2;4(5):e0002519. doi: 10.1371/journal.pgph.0002519 (PMC11065246; doi:10.1371/journal.pgph.0002519)
Supplement: Attachment — Submitted filename: Reviewer #4.docx [file pgph.0002519.s004.docx]

**Reviewer #4**

 11.	A good focus on the critical nutrient source (eggs) and effects on bones especially in older persons. the methods were good despite not getting enough papers for review. indicate that interpretation should be with caution. Following this review, the authors might consider a systematic review on the same topic to see of there is a wider scope of publications on the same topic, the authors have suggested the need for more studies, however, a systematic and or meta-analysis might also be useful

*Thank for your feedback. This is duly noted*.
