# Supplementary material for: Egg consumption and bone mass density among the elderly: A scoping review
Source: PLOS Glob Public Health. 2024 May 2;4(5):e0002519. doi: 10.1371/journal.pgph.0002519 (PMC11065246; doi:10.1371/journal.pgph.0002519)
Supplement: Attachment — Submitted filename: Reviewer #3.docx [file pgph.0002519.s005.docx]

**Reviewer #3**

Thank you for making the modifications to the manuscript. Its quality has improved. However, I believe some pertinent modifications are still required.

1.	Firstly, regarding the similarity analysis; excluding the published preprint, it is important to focus on the information presented in tables 1 and 2, as the objectives, conclusions, and other data are exactly like those in the cited article. I recommend condensing the information and avoiding the use of the same text as in the cited documents.

Response:

Thank you for your observation. The table content has been rephrased carefully (line 164).

“*This cross-sectional study presents evidence supporting a positive association between whole egg consumption and whole-body bone mineral density (WB-BMD). If future randomized controlled trials confirm these findings, incorporating whole eggs into the diets of older adults could have a substantial public health impact, including potential benefits for osteoporosis prevention and reduced fracture risk*.”

*“Approximately 56% of the participants in the study reported consuming between 1 to 5 eggs per week, with an average weekly intake of one egg.”*

*“This cross-sectional study presents evidence supporting a positive association between whole egg consumption and whole-body bone mineral density (WB-BMD). If future randomized controlled trials confirm these findings, incorporating whole eggs into the diets of older adults could have a substantial public health impact, including potential benefits for osteoporosis prevention and reduced fracture risk”*

*“The average daily intake for men was 20.2 grams, while for women it was 22.6 grams per day.”*

*“The WB T-score exhibited a positive correlation with daily egg consumption (r = 0.16; P = 0.027). Conversely, multiple fractures demonstrated a negative correlation with egg intake (r = -0.39; P = 0.014). In regression analysis, the WB T-score continued to show a positive association with egg intake (B = 0.02; P = 0.02).”*

*“There was no observed evidence indicating a correlation between egg consumption and the risk of hip fracture (OR = 0.99; 95% CI: 0.63, 1.56).”*

*“The study comprised a total of 646 case patients and 646 matched control subjects, of which there were 484 female pairs and 162 male pairs.”*

2.	Secondly, concerning the abstract, the results section can focus solely on the findings of this article. There is no need for a summary of the method; instead, the essence of the analysis findings is required.

*“While high egg intake has been linked to increased mortality and dyslipidemia, eggs contain compounds like ovotransferrin and carotenoids that may benefit bone health. As aging increases vulnerability to bone fragility and fractures, it's crucial to provide comprehensive dietary recommendations. The complex relationship between egg consumption, cholesterol, and health highlights the need for nuanced assessment. Overall, eggs present a potentially valuable dietary component for promoting bone health in aging populations.”*
